# Social well-being under pressure: structural inequities in early childhood in Mexico City

**DOI:** 10.3389/fpubh.2026.1702505

**Published:** 2026-03-18

**Authors:** Oscar A. Martínez-Martínez, Mónica Ancira-Moreno, Araceli Ramírez-López

**Affiliations:** 1 Universidad Iberoamericana, Research Center for Equitable Development (EQUIDE), México City, Mexico; 2 Universidad Iberoamericana, Departamento de Ciencias Sociales y Políticas, México City, Mexico; 3 Universidad Iberoamericana, Health Department, México City, Mexico; 4 Universidad Iberoamericana, Observatorio Materno Infantil, México City, Mexico; 5 Tecnológico de Estudios Superiores de Chicoloapan, Department of Computer Systems, México City, Mexico

**Keywords:** community wellbeing, early childhood, Mexico, objective wellbeing, social wellbeing, subjective wellbeing

## Abstract

**Introduction:**

Although progress has been made in measuring social wellbeing, limited evidence exists on its structure in households with young children living in contexts of structural inequality. This study asks: Which indicators most strongly shape social wellbeing in early childhood households?

**Methods:**

Using representative data from the 2024 Multidimensional Social wellbeing Survey, we applied the *DMM*−*RL* distance methodology, a multidimensional index construction technique that integrates objective and subjective indicators to compare the social welfare of households with and without children in early childhood.

**Results:**

Households with young children consistently showed lower wellbeing across most indicators. They had fewer economic resources, with a considerable share living in income poverty and lacking access to social security due to caregivers' concentration in informal employment. These households reported almost no leisure time, higher rates of depression, and limited community participation. Although food security, internet access, and basic services contributed positively, these advantages were insufficient to offset deficits in emotional and relational dimensions. Overall, structural disadvantages outweighed the compensatory effects of material resources.

**Discussion:**

Findings underscore that the social determinants of health intersect to produce multidimensional vulnerabilities in early childhood households. These results imply that policies focused primarily on income poverty may overlook structural constraints related to time scarcity, caregiver mental health, and weakened community ties. We recommend strengthening care and mental health support services, expanding access to social protection for caregivers in informal employment, and incorporating multidimensional wellbeing indicators—including time-use and psychosocial measures—into the design and monitoring of urban early childhood policy. By documenting these inequalities in an urban Global South context, the study provides evidence to guide more integrated health and social policy interventions to improve child and family wellbeing.

## 1 Introduction

Social wellbeing (SW) has evolved beyond a narrow focus on income or life satisfaction to encompass multiple dimensions of human life, including mental health, leisure time, and social cohesion, among others (1, 2). This reflects a shift from an individualistic to a more social or collective approach. Therefore, SW is conceived as a complex network of interrelated variables, influenced by both individual and societal factors (3).

This evolution has led to the adoption of multidimensional approaches to measuring SW, combining objective and subjective indicators that address micro- and meso-social dimensions (4, 5). Among these, health and education have been widely recognized as foundational pillars of SW (2, 6). Likewise, housing, not only as physical shelter but also as an environment that provides access to essential services such as drinking water, sewage systems, electricity, cooking fuel, and internet connectivity, is crucial for wellbeing (7).

Although the literature has made progress in identifying indicators to be integrated into SW measurement, research has mostly focused on the general population. Little is known about what occurs in specific vulnerable groups, such as households with children in early childhood (ECH), particularly in the Global South, as is the case in Mexico, where these households face adverse conditions—for example, 41.9% live in poverty and 8.8% in extreme poverty. In terms of social deprivation, 37.7% lack access to health services, 56.9% lack social security, and 19.3% face deficiencies in basic availability of services such as potable water, drainage, and overcrowding. Moreover, 16.1% experience food insecurity, and 15.1% have insufficient income to purchase food (8). These conditions of poverty and deprivation have lifelong consequences and contribute to the intergenerational transmission of poverty (9).

Early childhood is a critical window for the development of human potential, and ensuring SW during this stage is essential for laying the foundations of lifelong health, learning, and wellbeing. The first 1,000 days of life—from conception through a child's second birthday—are widely acknowledged as a sensitive period for optimizing health, nutrition, and neurodevelopment (10). Beyond this, the next 1,000 days (ages 2–5) are equally pivotal, as they shape brain architecture, emotional regulation, social competencies, and school readiness (11). Together, these two phases require sustained, holistic investments to promote wellbeing and ensure that all children achieve their full developmental potential.

In this context, the Nurturing Care Framework, developed by the World Health Organization (WHO), the United Nations Children's Fund (UNICEF), and the World Bank, proposes five interrelated components essential for optimal child development: health, nutrition, early learning, responsive caregiving, and security and safety (12). While these components provide a useful lens for examining and constructing indicators of social wellbeing in early childhood households, it is necessary to move toward multidimensional, contextual approaches that integrate not only objective indicators but also the cultural meanings that children and their caregivers assign to their daily lives (13, 14). These perspectives align with UNICEF Innocenti (15), which calls for integrated frameworks capable of addressing emerging global threats—such as pandemics, climate change, and digital transformation—that directly impact child wellbeing.

In highly urbanized countries such as Mexico, many of these structural inequalities are concentrated and intensified in large metropolitan areas. The present study focuses on Mexico City—the capital of Mexico and one of the largest metropolitan areas in Latin America—located in the central region of the country. Large urban centers concentrate infrastructure, labor markets, educational institutions, and public services, thereby enhancing certain objective dimensions of wellbeing. At the same time, urban density, spatial segregation, and socio-territorial inequality may generate heightened exposure to insecurity and stress (16). This configuration makes Mexico City an analytically relevant setting for examining how objective, subjective, and community dimensions of wellbeing may not necessarily evolve in parallel.

Although Mexico City reports lower multidimensional poverty levels than many other states in the country (8), it also records elevated levels of crime and perceptions of insecurity (17), as well as documented challenges in maintaining social cohesion across neighborhoods (18). These structural conditions allow for the exploration of how early childhood households experience wellbeing within a context characterized by both expanded service provision and persistent urban inequalities. Because many large metropolitan areas in middle-income countries share similar patterns of labor informality, spatial segregation, service stratification, and uneven access to social protection, the dynamics identified in Mexico City may resonate in other urban settings where objective advantages coexist with subjective and community-level stressors.

Understanding which indicators most strongly shape SW in households with young children has implications that extend beyond academic measurement debates. Identifying the relative contribution of objective, subjective, and community dimensions can inform the prioritization of public interventions, improve the design of early childhood policies, and help policymakers move beyond income-centered approaches toward more integrated strategies. A multidimensional perspective allows governments to detect hidden vulnerabilities—such as time poverty, caregiver mental health burdens, or limited social cohesion—that are often overlooked in traditional wellbeing metrics but may generate long-term social and economic costs.

Despite its importance, research on wellbeing in early childhood households remains limited. This study therefore seeks to fill this gap in the literature by addressing the following question: Which indicators contribute most to constructing SW in early childhood households?

## 2 Materials and methods

### 2.1 Measures

Social wellbeing comprises three dimensions: objective, subjective, and Community wellbeing. According to various studies (2, 4, 5), objective wellbeing (OWB) includes indicators related to household members' material conditions and access to basic household services. Subjective wellbeing (SWB) reflects individuals' Satisfaction in different areas of life. Community wellbeing (CWB) encompasses indicators of both material conditions and the social relationships that develop within a given context.

The integration of these three dimensions into a condensed measure is based on previous studies that have combined objective and subjective indicators at both micro- and meso-social levels to construct synthetic SW indices (7, 19). Table 1 presents the indicators for each of these dimensions, along with the methods used to calculate them.

**Table 1:** Structure and measures in the construction of the well-being index.

**Dimension**	**Indicator**	**Definition**	**Calculation method**
OWB	Education	Percentage of household members with no educational lag	A person has an educational gap if they fall into any of the following situations: (a) They are between three and fifteen years old, do not have compulsory basic education, and do not attend a formal school. (b) They were born before 1982 and did not complete compulsory primary education. (c) They were born after 1982 and did not complete compulsory secondary education (36)
	Food security	Indicator of households with food security	At the household level, levels of mild, moderate, or severe food insecurity are determined according to the Latin American and Caribbean Food Security Scale (ELCSA). Households not classified under these levels are considered food secure
	Quality of living spaces	Indicator of households with adequate housing quality and space	A dwelling with space deprivation is one that meets at least one of the following conditions: 1. The floor is made of dirt. 2. The roof is made of cardboard sheets or scraps. 3. The walls are made of mud or wattle and daub; reed, bamboo, or palm; cardboard, metal, or asbestos sheets; or scrap materials. 4. The overcrowding index is greater than 2.5. (36)
	Basic availability of basic services	Indicator of access to basic household services	A dwelling with a lack of basic availability of basic services is one that meets at least one of the following conditions: 1. There is no electricity. 2. The fuel used to cook or heat food is firewood or coal, and there is no chimney. 3. They do not have sewage service, or the sewer is connected to a pipe that leads to a river, lake, ocean, ravine, or crevice (36). 4. They do not have piped water in their home and experience water insecurity (37).
	Health	Percentage of household members with access to health services	A person has access to health services if they are affiliated with or entitled to receive medical services from an institution that provides them, such as: the Mexican social security institute (IMSS); the institute of security and social services for state workers (ISSSTE), either at the federal or state level; the Army or Navy; PEMEX; IMSS-BIENESTAR; or private medical services, or if they are a beneficiary of a social programs that includes medical care (36).
	Health perception	Self-rated health score	The self-perception of health rating is a standard variable and corresponds to the response to the question: In general, how would you rate your health status? Very good, Good, Fair, Poor, or Very poor (6).
	Depression	Percentage of individuals without depressive symptoms	Depression is determined using the score on the Center for Epidemiological Studies Depression Scale (38), which consists of 7 questions and ranges from 0–21 points.
	Anxiety	Percentage of individuals without anxiety symptoms	Anxiety is determined using the score on the anxiety scale (39), which consists of 7 questions and ranges from 7–28 points.
	Employment	Percentage of economically active household members	Percentage of household members belonging to the economically active population who worked the week prior to sampling.
	Social security	Percentage of household members with social security coverage	The population that does not meet any of the following criteria is considered to be deprived of access to social security (36). The economically active, salaried population that receives, through their work, the benefits established in Article 2 of the Social Security Law (or their equivalents in the legislation applicable to Section B of Article 123 of the Constitution). The self-employed or independent working population who receives medical services as a benefit from their employment or through voluntary recruitment into the mandatory IMSS system and who also have a retirement savings system (SAR) or Afore (Afore). The general population receives a retirement pension or other type of pension, or is a family member of a person inside or outside the household with access to social security (36). The population of retirement age (sixty-five years or older) who are beneficiaries of a non-contributory pension.
	Income	Percentage of household members without income deprivation	The percentage of household members who are not income-deprived is equal to: $\{\begin{array}{c} 100\%\;si\frac{ICT_{h}}{LB}\geq N_{h}\\ \frac{ICT_{h}\times 100\%}{N_{h}\times LB}\;si\frac{ICT_{h}}{LB}<N_{h} \end{array}$ Where *ICT*_*h*_ is the total current income of the household, equal to the sum of current monetary income and current non-monetary income. *N*_*h*_ is the number of household members according to equivalence scales, and *LB* is the income poverty line, that is, the food and non-food costs (36).
	Internet	Level of internet access at home	An ordinal variable indicating three levels of internet access: (a) lack: no internet access, neither via modem nor mobile data; (b) medium lack: limited access, only via mobile data; (c) no lack: internet access through a landline via modem.
	Internet access devices	Availability of internet access devices at home	An ordinal variable indicating three levels of internet access devices: (a) lack: when a computer, tablet, or laptop is available; (b) medium lack: when only a cell phone is available; (c) lack: when no access device is available.
	Cultural participation	Number of cultural events attended in the last twelve months	This is the number of times, during the past 12 months, the person attended community festivals and religious activities, dance performances, music concerts, museums, plays, archaeological sites, fine arts exhibitions, visual arts performances, historical monuments, cultural centers, national parks, or natural sites.
	Cultural consumption	Frequency of cultural product consumption	This is the score obtained from 5 questions, which gives a total range of 0–5 points. The questions ask about the following: Whether you read at least one book in the previous week. Whether you read an article, either in a magazine or online. Whether you read news, whether printed or online. Whether you listened to a debate or discussion program about the current situation in the country and the world. Whether you watched a television documentary about history, science, discoveries, art, trade, technology, nature, or travel.
	Leisure time	Percentage of people with sufficient leisure time	A person is experiencing leisure time deprivation if their number of leisure time hours falls below a threshold established according to their age group and educational status. The threshold is calculated as the total number of hours in a week, subtracting individual recovery time-which includes sleep, nutrition, personal care, rest, and health-related self-care- as well as time spent on domestic and extra-domestic work, and study time, including commuting (40).
SWB	Satisfaction in different domains of life	Score on life satisfaction across different domains	The satisfaction scale scores measures, on a scale of 0–10, satisfaction across various aspects: life in general, social life, family life, emotional life, standard of living, personal achievements, neighborhood, economic situation, and the health status of household members.
CWB	Social cohesion	Synthetic social cohesion index (micro, meso, macro levels)	A synthetic indicator that includes the micro, meso, and macro-social levels: The micro level includes the willingness to help groups of friends and groups of belonging, such as religious, sports, cultural, and artistic, among others. The meso level encompasses (a) ties with neighborhood members, as well as the norms and values that predominate in that community; (b) willingness to help neighbors; (c) participation in solving community problems. The macro level focuses on trust in institutions (41).
	Built environment	Level of neighborhood walkability	A synthetic indicator of the Neighborhood Walkability Scale (NEWS), adapted for Mexico, which measures accessibility to various commercial establishments, street lighting, availability of green areas, quality of sidewalks, presence of pedestrian crossings, and traffic lights (42).
	Quality of public transportation	Perceived quality of public transportation (rating scale)	The respondent's rating of the quality of public transportation used is based on a five-point Likert scale: very good, good, fair, poor, and very poor.
	Perception of insecurity	Score on the perception of insecurity scale (neighborhood, municipal, city)	This is the score for three questions: do you consider living in your (a) neighborhood; (b) municipality; (c) city to be: very safe, safe, neither safe nor unsafe, unsafe, or very unsafe? The assigned score ranges from 0 points for very unsafe to 4 points for very safe.
	Protection from crime	Percentage of household members not victimized by crime	Proportion of common crimes from which household members have been safe in the past year. These crimes include vehicle theft, vandalism of private property, robbery, bank fraud, extortion, verbal threats, kidnapping, physical assault, and enforced disappearance.

Source: author's elaboration.

### 2.2 Sampling

**Figure 1:**
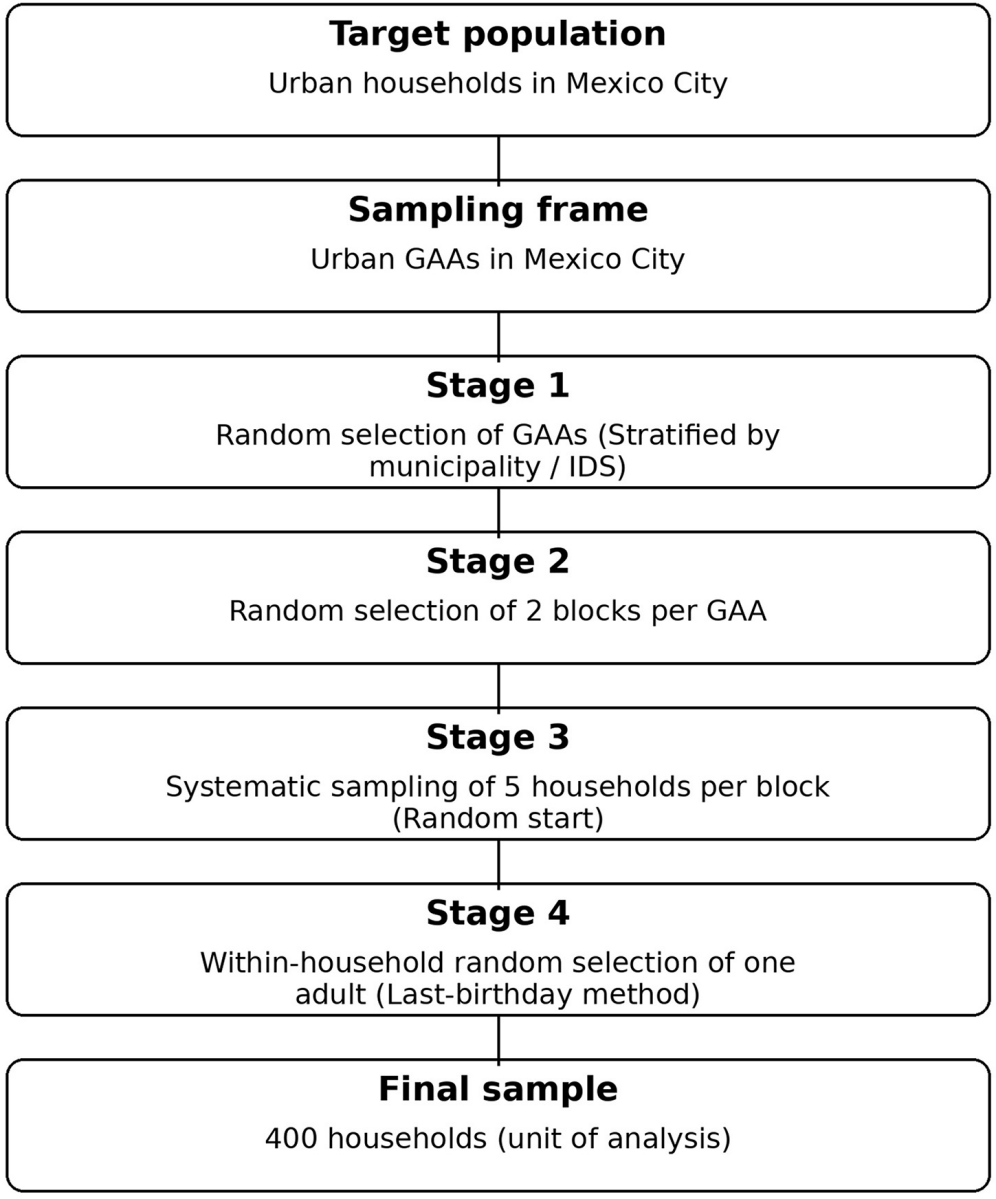
Multistage probabilistic sampling design of the MSWS 2024. Source: author's elaboration.

Data were drawn from the 2024 Multidimensional Social wellbeing Survey (MSWS) (20), which is representative of Mexico City. The sampling design was probabilistic, stratified, and cluster-based, with the city's 16 municipalities sampled proportionally to their population sizes. The MSWS was conducted in four stages: (a) Geographic Assessment Areas (GAAs) were randomly selected within each municipality, based on levels of social development as measured by the Mexico City government's Social Development Index (Índice de Desarrollo Social, IDS) (21); (b) two blocks or survey points were randomly selected within each selected GAA; (c) five households per block were selected using systematic sampling with a random start; and (d) within each household, one adult respondent was randomly selected using the last-birthday method—that is, the adult whose birthday occurred most recently—and asked to provide informed consent before survey administration (see Figure 1). The household served as the primary unit of analysis.

Eligibility criteria were as follows: (i) households located within the selected GAAs; (ii) occupied private dwellings; (iii) households with at least one adult (≥18 years old) present and available for interview at the time of the visit; and (iv) respondents who provided informed consent. Exclusion criteria included: (i) vacant dwellings or non-residential buildings; (ii) collective dwellings (e.g., shelters or institutions); (iii) households in which no eligible adult was present after repeated visits; and (iv) households that declined participation.

The average survey duration was approximately 1.5 h, and a total of 400 questionnaires were completed, yielding a response rate of 97%. The sample size is consistent with the conventional formula for estimating population proportions under simple random sampling:


$$\begin{array}{l} n=\frac{Z^{2}\;p\left(1-p\right)}{e^{2}} \end{array}$$

where *Z* = 1.96 corresponds to a 95% confidence level, *p* = 0.5 is a conservative assumption that maximizes variance in the absence of prior information on the population proportion, and *e* = 0.049 is the target margin of error. Substituting these values yields *n*≈400. Although the design was a multistage cluster sample, the reported margin of error is based on a simple random sampling approximation and does not explicitly account for the design effect.

Population expansion factors were then calculated based on selection probabilities and adjusted using a ranking calibration algorithm to correct for deviations in age and sex distributions. Finally, the weights were normalized through weight trimming. The study was approved by the Ethics Committee under protocol number CEI: 8/23.

### 2.3 Procedure and data analysis

The methodology for calculating the wellbeing index is initially based on the *DP*2 distance method (22), which has been widely applied for this purpose (23, 24). However, given the nature of our data—which includes both objective and subjective variables—we developed the *DMM*−*RL* method, which explicitly accounts for the measurement scale of each indicator, whether continuous, ordinal, or binary.

To implement the methodology, we define *X* = {*x*_*ij*_} the data matrix corresponding to *m* indicators (columns) associated with *n* sample elements (rows), where *x*_*ij*_ represents the value of the *j*-th indicator for the *i*-th sample element. The indicators can be measured on continuous, ordinal, or binary scales. The synthetic wellbeing index for the *i*-th household is given by:


$$\begin{array}{l} {\left(DMM-RL\right)}_{i}=\overset{m}{\underset{j=1}{\sum }}\frac{d_{ij}}{\sigma_{j}}w_{j}\left(1-R_{j,j-1,j-2,..,1}^{2}\right);\;i=1,2,\;\ldots ,n. \end{array}$$

where $d_{ij}=|x_{ij}-{x_{pj}}^{*}|$ is the distance between the *j*-th indicator of the *i*-th household and the minimum value of the *j*-th indicator in the data matrix, *x*_pj_^*^.

σ_*j*_ is a measure of the variability of the data for the *j*-th indicator, and its calculation depends on the indicator scale. For continuous indicators, σ_*j*_ the sample standard deviation of the *j*-th indicator. For binary indicators, $\sigma_{j}=\sqrt{p_{j}\left(1+p_{j}\right)}$, where *p*_*j*_ is the proportion of ones in the *j*-th indicator (25). And for ordinal indicators with *r* levels, $\sigma_{j}=-\overset{k}{\underset{r=1}{\sum }}p_{j}^{r}ln\left(p_{j}^{r}\right)$, where $p_{j}^{r}$ is the proportion of observations corresponding to the level *r* of the *j*-th indicator (26).

The $R_{j,j-1,j-2,..,1}^{2}$ term is the coefficient of determination in the regression of the *j*-th indicator (dependent variable) on the indicators *j*−1, *j*−2, …, 1 (explanatory variables). When the dependent variable is continuous, $R_{j,j-1,j-2,..,1}^{2}=1-\frac{SSR}{SST}$, where *SSR* is the sum of squared residuals of the model and *SST* is the total sum of squared differences between the observed values and their mean. For binary or ordinal dependent variables, $R_{j,j-1,j-2,..,1}^{2}=1-{\left(\frac{L_{modelo}}{L_{nulo}}\right)}^{2/n}$, where *L*_*modelo*_ is the likelihood of the fitted model and *L*_*nulo*_ is the likelihood of the null model (25). It is important to note that $R_{j,j-1,j-2,..,1}^{2}$ takes values between 0 and 1 in all cases. Additionally, it is assumed that $R_{1}^{2}=1$. The correction factor $\left(1-R_{j,j-1,j-2,..,1}^{2}\right)$ eliminates redundant information by accounting for potential interdependencies among the indicators.

*w*_*j*_ is the exogenous weight assigned to the *j*-th indicator. This weight reflects the indicator's importance in the index construction and ranges from 0 to 1, where 0 indicates the indicator is irrelevant and 1 indicates high importance.

The synthetic index (*DMM*−*RL*)_*i*_ for the household *i* is the sum of the contributions of each indicator to wellbeing. The contribution of the *j*-th indicator is determined by the standardized distance between that indicator and its minimum value; this distance is then adjusted to account for redundant information present in the indicator, as well as the exogenously assigned weight to reflect its importance.

It is worth noting that, given a sample *X* = {*x*_*ij*_}, the algorithm can be applied to the entire sample (i.e., for all *i* = 1, 2, …, *n*) or to specific subgroups within it. In this study, we consider both the full sample and two subgroups: the first consists of households with children aged 0–5 years, and the second consists of households without children in that age range.

Finally, the method described is applied in conjunction with the bootstrap sampling technique, whereby the procedure is repeated on random samples drawn from the original dataset. This enables the estimation of confidence intervals for both the mean of the wellbeing index and the average contribution of each indicator across different household groups. This approach yields more robust estimates of the variability and precision of the estimators, providing a more realistic depiction of the dispersion of wellbeing across subgroups. Its utility is particularly relevant when the data exhibit skewness or contain outliers, as the bootstrap method does not rely on strict distributional assumptions (27, 28).

All data processing and statistical analyses were conducted in R (version 4.3.1), using custom scripts to implement the *DMM*−*RL* algorithm and the bootstrap procedure.

## 3 Results

Table 2 shows the percentage coverage of SW indicators, first for all households and then disaggregated into early childhood households (ECH households) and households without children in early childhood (non-ECH households). A high proportion of households report favorable conditions across several wellbeing indicators, including quality of living spaces (98.66%), employment (98.49%), education (94.72%), internet access (90.24%), and food security (82.40%). In contrast, only 62.67% report sufficient income to cover food and basic household expenses, 62.01% have access to health services, and 57.73% are covered by social security.

**Table 2:** Percentage of coverage of the indicators.

**Indicator**	**% of all households**	**% ECH**	**% non-ECH**
Quality of living spaces	98.66	98.01	98.73
Employment	98.49	99.59	98.37
Education	94.72	87.69	95.49
Internet	90.24	98.09	89.39
Internet access devices	83.32	90.79	82.51
Food security	82.40	89.31	81.65
Anxiety	82.19	75.69	82.90
Protection from crime	77.22	70.07	78.00
Health perception	75.12	92.87	73.18
Basic availability of basic services	71.56	73.18	71.39
Satisfaction in different domains of life	64.05	62.91	64.18
Quality of public transportation	63.29	72.07	62.33
Depression	63.01	54.84	63.89
Income	62.67	61.23	62.90
Health	62.01	54.95	63.19
Social security	57.73	52.34	58.64
Cultural consumption	50.69	44.68	51.35
Built environment	46.40	64.58	44.41
Cultural participation	45.04	42.35	45.34
Perception of insecurity	38.26	38.65	38.22
Social cohesion	35.91	30.19	36.54
Leisure time	2.28	0.90	2.53

Source: author's elaboration.

Regarding mental health, 82.19% of household members reported no symptoms of anxiety, and 63.01% did not report symptoms of depression. Additionally, 75.12% perceived their health status as good or very good. Regarding public safety, 77.22% had not been victims of crime. By contrast, only 38.26% perceived their neighborhood, municipality, or city as safe or very safe. This perception may partly explain why only 35.91% believed that social cohesion exists. A notable finding is that only 2.28% of respondents reported having leisure time available.

When comparing indicators between ECH and non-ECH households, percentage differences were generally small, particularly for indicators with the highest overall coverage, such as quality of living spaces and employment. ECH households show higher coverage in indicators such as internet access and digital devices, food security, self-perceived health, availability of basic services, quality of transportation, built environment conditions, and perception of insecurity. In contrast, non-ECH households exhibit higher levels of education, protection from crime, income, access to health services, and social security. They also report greater satisfaction across life domains—including consumption and cultural participation—higher levels of social cohesion, and greater availability of leisure time. In addition, this group shows a lower prevalence of anxiety and depression symptoms.

Table 3 presents the results obtained using the *DMM*−*RL* method. All indicators across the three wellbeing dimensions contributed significantly to the construction of SW, as indicated by their 95% confidence intervals. Although the magnitude of each contribution varied (see last columns), the indicators with the highest average contributions were employment, followed by quality of living spaces, education, and protection from crime. In contrast, leisure time showed the lowest average contribution.

**Table 3:** Mean and confidence intervals of the contribution of the indicators to the social wellbeing index by type of household.

**Indicator**	**ECH**		**non-ECH**		**Total**	
	**Mean (SD)**	**Intervals**	**Mean (SD)**	**Intervals**	**Mean (SD)**	**Intervals**
^a^Food security	1.80 (0.15)	[1.78, 1.81]	1.64 (0.13)	[1.63, 1.66]	1.66 (0.13)	[1.65, 1.67]
^a^Internet	1.81 (0.25)	[1.79, 1.83]	1.70 (0.24)	[1.68, 1.72]	1.71 (0.24)	[1.69, 1.73]
^a^Internet access devices	1.63 (0.12)	[1.62, 1.64]	1.54 (0.11)	[1.53, 1.55]	1.55 (0.11)	[1.54, 1.56]
^a^Basic availability of basic services	1.30 (0.19)	[1.29, 1.32]	1.27 (0.08)	[1.27, 1.28]	1.28 (0.08)	[1.27, 1.28]
^a^Health perception	0.66 (0.06)	[0.65, 0.66]	0.63 (0.06)	[0.62, 0.63]	0.63 (0.06)	[0.63, 0.64]
^b^Built environment	0.61 (0.08)	[0.61, 0.62]	0.53 (0.05)	[0.52, 0.53]	0.54 (0.05)	[0.53, 0.54]
^b^Quality of public transportation	0.56 (0.05)	[0.55, 0.56]	0.54 (0.04)	[0.54,0.55]	0.54 (0.04)	[0.54, 0.55]
^b^Perception of insecurity	0.16 (0.04)	[0.16, 0.17]	0.13 (0.01)	[0.13, 0.14]	0.14 (0.01)	[0.14, 0.15]
^a^Education	4.83 (0.63)	[4.78, 4.89]	5.28 (0.66)	[5.22, 5.33]	5.23 (0.65)	[5.17, 5.29]
^b^Protection from crime	4.22 (0.85)	[4.14, 4.29]	4.37 (0.84)	[4.29, 4.44]	4.35 (0.84)	[4.28, 4.43]
^a^Income	1.54 (0.20)	[1.53, 1.56]	1.57 (0.09)	[1.56, 1.57]	1.56 (0.09)	[1.56, 1.57]
^a^Health	1.16 (0.22)	[1.14, 1.18]	1.32 (0.07)	[1.31, 1.32]	1.30 (0.07)	[1.30, 1.31]
^a^Social security	0.60 (0.13)	[0.59, 0.61]	0.60 (0.07)	[0.60, 0.61]	0.60 (0.07)	[0.60, 0.61]
^b^Social cohesion	0.30 (0.06)	[0.30, 0.31]	0.36 (0.04)	[0.35, 0.36]	0.35 (0.04)	[0.35,0.36]
^a^Cultural consumption	0.26 (0.08)	[0.25, 0.26]	0.33 (0.02)	[0.32, 0.33]	0.32 (0.02)	[0.32,0.33]
^a^Cultural participation	0.09 (0.03)	[0.09, 0.10]	0.19 (0.02)	[0.19, 0.20]	0.18 (0.02)	[0.18, 0.19]
^a^Leisure time	0.01 (0.01)	[0.01, 0.02]	0.14 (0.09)	[0.13, 0.15]	0.13 (0.10)	[0.12, 0.14]
^a^Employment	8.14 (1.57)	[8.00, 8.28]	8.03 (1.57)	[7.89, 8.17]	8.04 (1.57)	[7.90, 8.18]
^a^Quality of living spaces	7.16 (1.74)	[7.01, 7.31]	7.20 (1.74)	[7.05, 7.36]	7.20 (1.74)	[7.05, 7.35]
^a^Depression	2.77 (0.36)	[2.73, 2.80]	2.80 (0.36)	[2.77, 2.84]	2.77 (0.35)	[2.74, 2.80]
^c^Satisfaction in different domains of life	1.74 (0.20)	[1.72, 1.75]	1.75 (0.20)	[1.74, 1.77]	1.75 (0.20)	[1.73, 1.77]
^a^Anxiety	2.06 (0.29)	[2.03, 2.08]	2.08 (0.29)	[2.05, 2.10]	2.07 (0.29)	[2.05, 2.10]
**Social well-being index**	**43.4 (2.63)**	**[43.17, 43.63]**	**43.95 (2.63)**	**[43.72, 44.18]**	–	

Source: author's elaboration.

^a^OWB.

^b^CWB.

^c^SWB.

A second key finding is the synthetic SW index and its 95% confidence intervals, which indicate that non-ECH households exhibit higher overall wellbeing than ECH households. This difference is explained by the differential average contributions of each indicator, with some indicators demonstrating greater weight and statistical relevance depending on household type. These results address the research question by identifying the indicators that most strongly shape SW in ECH households.

The first indicator listed in Table 3 is food security, with an average contribution of 1.80 in ECH households, higher than that observed in non-ECH households (1.64). The second and third indicators relate to technology access: internet connectivity contributes an average of 1.81 in ECH households vs. 1.70 in non-ECH households, while availability of internet-enabled devices contributes 1.63 compared to 1.54, respectively. The availability of basic household services contributes 1.30 in ECH households and 1.27 in non-ECH households. Regarding self-perceived health status, the contribution is also higher in ECH households (0.66) than in non-ECH households (0.63).

In the built environment, ECH households show a marginally higher average contribution (0.61) than non-ECH households (0.53). A similar pattern is observed for transportation quality (0.56 vs. 0.54). Perception of insecurity contributes 0.16 in ECH households, compared to 0.13 in non-ECH households.

In contrast, non-ECH households exhibit higher contributions in education (5.28 vs. 4.83), protection from crime (4.37 vs. 4.22), and income (1.57 vs. lower values in ECH households). Access to health services also yields higher contributions in this group (1.32 vs. 1.16).

Other indicators—such as social cohesion, cultural consumption, and cultural participation—also register higher contributions among non-ECH households. Social cohesion contributes 0.36, compared to 0.30; cultural consumption, 0.33, vs. 0.26; and cultural participation, 0.19, vs. 0.09. Although leisure time has the lowest average contribution to SW overall, its value remains higher in non-ECH households (0.14) than in ECH households.

Some indicators did not show statistically significant differences in their average contributions between household types, suggesting similar influence across groups. These include employment (8.04), quality of living spaces (7.20), depression (2.77), satisfaction across life domains (1.75), anxiety (2.07), and social security (0.60). All of these indicators made meaningful contributions to the SW index, with employment and quality of living spaces standing out as particularly influential.

## 4 Discussion

Social wellbeing results from the interaction between objective and subjective indicators across multiple dimensions. These dimensions operate simultaneously and reinforce each other, as evidenced by the data. The findings confirm that social wellbeing is not only an individual experience but also a collective one, embedded in broader micro- and meso-social contexts. In this sense, subjective indicators provide insight into affective, evaluative, and contextual dimensions of child wellbeing that are often overlooked by traditional metrics (13).

These dimensions reflect how children and caregivers interpret their living conditions. Child wellbeing should therefore not be understood as a linear outcome of structural inputs, but as a process shaped by relational bonds, symbolic opportunities, and capabilities developed in interaction with their surroundings (14). This is particularly relevant for early childhood households (ECH households), whose wellbeing depends heavily on the economic and social conditions of the home, placing them in structurally vulnerable positions. This vulnerability is compounded by the fragility of protection systems for children, a weakness further exposed by global crises such as the COVID-19 pandemic and recent economic downturns (15).

Although all indicators contributed to social wellbeing, their contributions varied substantially. This heterogeneity reflects differentiated capacities to realize rights and opportunities, consistent with national policy frameworks such as the Estrategia Nacional de Atención a la Primera Infancia (ENAPI) (29). From a policy perspective, this variation highlights the uneven territorial and institutional capacity to operationalize comprehensive early childhood interventions. Indicators with the lowest contributions point to areas where policy interventions may be required, particularly leisure time, cultural consumption, and cultural participation. Rather than reflecting marginal dimensions of wellbeing, these low contributions may signal implementation gaps in domains that are formally recognized but weakly operationalized within existing policy frameworks.

When comparing household types, most indicators were statistically significant, although their relative contributions differed. In ECH households, the most influential indicators included food security, access to digital devices, availability of basic services, perceived health status, built environment conditions, transportation quality, and perceptions of insecurity. This pattern reflects the centrality of material and environmental conditions in shaping wellbeing during early childhood, particularly in urban and socioeconomically unequal contexts.

Food security emerges as a central determinant of wellbeing among ECH households. This finding aligns with evidence positioning early childhood nutrition as a foundational component of child development and long-term wellbeing (10, 30). Although not directly tested in this study, this pattern may be associated with the reach of targeted social programs. In the sample, 51.43% of ECH households reported receiving the Support for the wellbeing of Children of Working Mothers program, 64.71% received the LICONSA Social Milk Supply program, and 68.77% received the My Scholarship to Start (Preschool) program. These programs may help explain the relative strength of food security contributions, illustrating how targeted social protection mechanisms can enhance specific domains of wellbeing.

Health perceptions also contribute more strongly in ECH households, reinforcing the centrality of health in contexts with young children. This likely reflects both caregiving demands and the heightened health needs of young children. ENAPI emphasizes the integral nature of child wellbeing, including maternal and caregiver health as essential components of nurturing environments (29). This is consistent with the global Nurturing Care Framework, which identifies health, nutrition, safety, responsive caregiving, and early learning as critical components of early childhood development (12). From a policy standpoint, these results suggest that strengthening maternal and caregiver health services may generate multiplier effects on child wellbeing being by enhancing both objective conditions and subjective perceptions of security and care (31).

At the same time, households without young children (non-ECH households) show higher contributions in education, income, social security, and access to health services. These differences suggest greater economic stability and labor market attachment. Many caregivers in ECH households are employed informally, limiting access to employment-linked social protection and reinforcing income constraints. This dual burden of insufficient income and limited social protection contributes to persistent intergenerational vulnerability (32), consistent with evidence linking poverty and social exclusion with lower perceived wellbeing in early childhood contexts (33). These findings underscore the importance of policy designs that decouple access to social protection from formal labor market participation, particularly for households with young children.

Indicators with the lowest contributions—particularly leisure time—reveal structural pressures. Nearly all ECH households reported lacking available leisure time, suggesting substantial time burdens associated with caregiving. International evidence indicates that caregivers in vulnerable contexts frequently face trade-offs between caregiving and self-care, limiting rest, recreation, and social participation (34). This form of time poverty represents a structural constraint that is rarely addressed explicitly in social policy design. This time, poverty may contribute to caregiver burnout and may help explain the elevated prevalence of depressive symptoms observed in these households. Since caregiver mental health is a known predictor of child resilience and adaptive capacity (35), the combination of time scarcity and psychosocial stress presents a significant structural risk. These findings point to the need for care-sensitive and time-sensitive policy approaches that recognize caregiving as a central determinant of child wellbeing.

Cultural participation and consumption also contribute minimally, reflecting constrained time and financial resources. From a rights-based perspective promoted in ENAPI, access to culture and recreation forms part of comprehensive early childhood development and should not be treated as secondary (29). Ensuring these rights in urban settings, such as Mexico City, requires policy approaches that go beyond material provision.

Community-level indicators also warrant attention. High levels of perceived insecurity in Mexico City—a large metropolitan area characterized by significant socio-spatial inequalities—reduce the positive contribution of safety-related indicators to overall wellbeing. ECH households report higher exposure to crime victimization, which may intensify stress and reduce social participation. Similarly, lower levels of social cohesion indicate limited neighborhood engagement and mutual support. The demands of caregiving may restrict opportunities for community involvement, weakening relational networks that typically sustain collective wellbeing.

Overall, these patterns reveal structural differences in wellbeing across household types. In large metropolitan areas such as Mexico City, material advantages do not necessarily translate into parallel improvements in subjective or community wellbeing. This gap underscores the need for integrated, territorially grounded early childhood policies that address material, relational, and psychosocial dimensions of wellbeing simultaneously.

## 5 Conclusion

Social wellbeing among ECH households reveals a multidimensional structure of structural vulnerability characterized by time scarcity, limited cultural engagement, lower social cohesion, and heightened exposure to mental health challenges. Although these households show strengths in food security, internet access, and basic services—likely reflecting the reach of existing public programs—these advantages do not offset accumulated disadvantages in subjective and community domains.

These findings have concrete implications for policy design in Mexico City and comparable metropolitan areas in middle-income countries. Public interventions should move beyond income transfers alone and integrate three specific priorities: (1) expanding accessible and affordable childcare services to reduce time poverty; (2) strengthening community-based mental health services for caregivers; and (3) extending social protection coverage to informal workers, particularly those with young children. Incorporating multidimensional wellbeing indicators—including time-use, psychosocial health, and community engagement—into monitoring systems can improve targeting and evaluation of early childhood programs.

These recommendations are particularly relevant for early childhood policymakers, urban planning authorities, public health agencies, and social protection institutions responsible for reducing structural inequalities in metropolitan contexts. By identifying the indicators that most strongly shape wellbeing in ECH households, this study provides actionable evidence by jointly analyzing objective, subjective, and community-level indicators in a large metropolitan context to prioritize interventions and refine urban early childhood policy strategies.

Finally, some limitations must be acknowledged. Although the sample is representative of Mexico City, it is not representative at the municipal level, where heterogeneity may exist. In addition, the cross-sectional design limits causal inference. Overall, these findings are consistent with ENAPI's objectives, reinforcing the need for integrated, intersectoral, and territorially grounded early childhood policies that address material conditions, caregiving constraints, and psychosocial dimensions of wellbeing in urban contexts. Nonetheless, the study offers a robust multidimensional framework for analyzing social wellbeing in early childhood households and contributes to ongoing debates on urban inequality and child vulnerability in the Global South.

## Data Availability

The data sets analyzed for this study are available on request from the corresponding author.
